# Generalizing About Striking Properties: Do Glippets Love to Play With Fire?

**DOI:** 10.3389/fpsyg.2019.01971

**Published:** 2019-08-29

**Authors:** Dimitra Lazaridou-Chatzigoga, Napoleon Katsos, Linnaea Stockall

**Affiliations:** ^1^Department of English and American Studies, Humboldt University of Berlin, Berlin, Germany; ^2^Department of Theoretical and Applied Linguistics, University of Cambridge, Cambridge, United Kingdom; ^3^Department of Linguistics Queen Mary, University of London, London, United Kingdom

**Keywords:** generic language, generalization, inference, language acquisition, striking property

## Abstract

Two experiments investigated whether 4- and 5-year-old children are sensitive to whether the content of a generalization is about a salient or noteworthy property (henceforth “striking”) and whether varying the number of exceptions has any effect on children’s willingness to extend a property after having heard a generalization. Moreover, they investigated how the content of a generalization interacts with exception tolerance. Adult data were collected for comparison. We used generalizations to describe novel kinds (e.g., “glippets”) that had either a neutral (e.g., “play with toys”) or a striking property (e.g., “play with fire”) and measured how willing participants were to extend the property to a new instance of the novel kind. Experiment 1 demonstrated that both adults and children show sensitivity to strikingness in that striking properties were extended less than neutral ones, although children extended less than adults overall. The responses of both age groups were significantly different from chance. Experiment 2 introduced varying numbers of exceptions to the generalization made (minimal: 1 exception; maximal: 3 exceptions). Both adults and children extended both types of properties even in the face of exceptions, but to a lower degree than in Experiment 1. Striking properties were extended less than neutral ones, as in Experiment 1. We observed that the greater the number of exceptions, the lower the rates of extension we obtained, for both types of properties in adults, but only with striking properties in children. Children seemed to keep track of varying numbers of exceptions for striking properties, but their performance did not differ from chance. The findings underscore that 4- and 5-year-old children are sensitive to strikingness and to exception tolerance for generalizations and are developing toward an adult-like behavior with respect to the interplay between strikingness and exception tolerance when they learn about novel kinds. We discuss the implications of these results with regards to how children make generalizations.

## Introduction

Children acquiring language and knowledge about the world are faced with a challenge when they encounter a specific object: which properties of that object are general properties of all objects of the same kind, and which are unique to that object. For example, a child seeing a black cat that has whiskers should generalize that cats have whiskers, but not draw the inference that cats are black. This is the case both for obvious properties of kinds, (e.g., “cats have whiskers”), which can be acquired through direct perceptual experience, but also for imperceptible ones (e.g., “cats are curious”). Properties can furthermore be common and familiar as in “cats have whiskers” or salient or noteworthy (henceforth “striking”) like “sharks attack people.” Furthermore, children have to pay attention to the possibility of exceptions to a generalization they have formed. That is, the occurrence of a whiskerless cat should not make them revise their generalization that cats have whiskers. In two experiments, we investigated whether 4- and 5-year-old children are sensitive to whether the content of a generalization is about a striking property and to how the content of a generalization interacts with exception tolerance.

Turning to the language used to convey information that belongs to kinds of objects rather than to individual instances thereof, in these investigations we use *generic statements* of the type “cats have whiskers,” “birds fly” or “sharks attack people.” Children show remarkable sensitivity to the distinction between generic and non-generic (specific, quantificational) nominals (Gelman et al., 2002, 2015; Gelman and Raman, 2003; Chambers et al., 2008 among others).

For example, numerous studies by Gelman and colleagues argue that preschoolers understand generics and are able to distinguish them from non-generics on the basis of lexical, morphosyntactic and contextual cues (see Gelman, 2003, 2010 for an overview and Lazaridou-Chatzigoga et al., 2015 for a critical review). Children seem to interpret bare plural generics as different from both statements with “some” and statements with “all.” For instance, in Gelman et al.’s (2002) study, children treated generics as intermediate between “all” and “some.” Children seem to rely on generics when they learn about familiar and novel kinds and they use them in order to draw inferences (Hollander et al., 2002; Gelman and Bloom, 2007). Children seem to know that a property introduced with a generic is likely to be extended to new instances of the kind, both when they learn novel properties of familiar categories (Gelman et al., 2002; Chambers et al., 2008 with 4-year-olds) and when they learn properties of novel kinds (Graham et al., 2011 with 30-month-old children).

However, the above evidence that children comprehend certain aspects of the meaning of generics by age 4 does not mean that children know how to integrate different types of properties (neutral vs. striking) in their conceptualization of kinds when they form generalizations. Additional features that need to be learned include: (a) the fact that generalizations are not only about *characteristic* properties, such as having whiskers or nursing young, but can also be about *striking* properties that are sufficiently noteworthy to allow generalization, even in the absence of strong statistical prevalence (i.e., “sharks attack people” is a felicitous generalization even though very few of the world’s population of sharks do so) and (b) the fact that generalizations allow for varying numbers of exceptions (“cats have whiskers” allows for a few accidental exceptions of whiskerless cats, “cats nurse their kittens” allows for a wide range of systematic exceptions, namely, immature cats and male cats, and “sharks attack people” allows for the vast majority of sharks to be exceptions). These two features might be challenging for children, as they force them to be attentive not only to the language used to generalize (generic or not), but also to the property that is predicated of the kind as well as to the possibility of the generality not holding for all instances of a kind, but only for most or some instances thereof.

There is some indicative evidence that children understand that generalizations tolerate exceptions (see discussion in the next section), but whether children know how to integrate information about varying numbers of exceptions and how children use that information while learning about novel kinds has not been investigated before. Furthermore, the majority of the studies thus far have focused on neutral properties without looking at properties that are noteworthy, distinctive or striking in some way. The present studies focus on how children draw generalizations and on how type of property and the varying number of exceptions interact. Before describing our studies, we provide a short overview of the relevance of type of property and exception tolerance for generalizations.

## Two Characteristics of Generalizations: Type of Property and Exceptions

### Type of Property

Prototypical generalizations such as “cats meow” and “tigers have stripes” communicate strong generalizations and are about a highly prevalent property of a kind.

Carlson (1977, p. 40) was the first to notice that examples like “mosquitoes carry the paramecium that causes yellow fever” are true of only a minority of the individuals under consideration, but the sentence seems felicitous nevertheless. These so-called “troublesome” generic generalizations have recently attracted the attention of psychologists (Leslie, 2007, 2008) and have been called “striking” generalizations (Leslie et al., 2011; Prasada et al., 2013). They are characterized as cases where the “property must only be had by a small minority of the kind, and must signify something dangerous and to be avoided” (Prasada et al., 2013, p. 409; Leslie, 2007, 2008, 2017). Prasada (2010) argues that striking generalizations involve *causal connections* between the kind and the property, in the sense that there is something about the nature of the individuals that form the kind that causes them to be disposed to have the property in question, irrespectively of whether they actually possess it or not. To illustrate, “mosquitoes carry the West Nile virus” does not mean that the majority of mosquitoes carry the West Nile virus, but that there is something in their common biological structure that causes them to have the disposition to carry the disease. For more discussion, see also Sterken (2015), where other proposals about how to deal with these generalizations are discussed.

Leslie et al. (2011) compare striking generalizations to a number of other types of generalization such as “quasi-definitional” as in “triangles have three sides” (which are exceptionless), “majority characteristic” as in “dogs have four legs” (which are true of the overwhelming majority of instances, with only a few exceptional individuals), “minority characteristic” as in “ducks lay eggs” (which involve primary or secondary sexual characteristics of animals, and are thus only true of no more than 50% of individuals) and “false generalizations” as in “*books are* paperbacks” (which may be true of 80% or more of individuals, and yet are not typically judged as true, and thus fail as generic statements).

An interesting question arises with respect to striking generalizations irrespective of the theoretical account we adopt to analyze them: Are striking generalizations accepted despite having low prevalence estimates? In the few relevant studies (Leslie et al., 2011; Prasada et al., 2013) adult participants accepted striking generalizations of the type “sharks attack people” at rates between 70 and 77%. These rates were lower than acceptance rates of quasi-definitional statements like “ants are insects” (90%) and majority characteristic statements like “tigers have stripes” (96%) and somewhat lower but still similar to minority characteristic statements like “ducks lay eggs” (85 to 92%). They differed more clearly from acceptance rates for false generalizations like “books are paperbacks” (38%), which were mostly rejected.

Leslie (2008) argues that people are biased to attend to properties that are striking or pose a threat to them, even when their prevalence in the kind in question is not high. Cimpian et al. (2010) reasoned that, based on Leslie (2008), this bias may manifest as a tendency to accept generalizations about striking properties more than about similar properties that do not have these connotations. In a truth conditions task, the participants were first given information about a property of a new kind at different prevalence levels (10, 30, 50, 70, and 90%) in one of three forms: (a) plain (“xx% of morseths have silver fur”), (b) dangerous/distinctive (“xx% of morseths have dangerous silver fur. This fur sheds particles that get logged in your lungs and make it impossible to breathe. No other animals on this island have this kind of fur”) or (c) non-distinctive control (“xx% of morseths have curly silver fur. This fur is very curly and rough to touch. Other animals on this island also have this kind of fur”). The participants were then asked whether the following sentence was true or false in either the generic (“morseths have silver fur/dangerous silver fur/curly silver fur”) or the *most-*form (“most morseths have silver fur”/dangerous silver fur/curly silver fur”). In an implied prevalence task, the participants first read a sentence in either the generic (“morseths have silver fur/dangerous silver fur/curly silver fur”) or the *most-*form (“most morseths have silver fur”/dangerous silver fur/curly silver fur”) and they then were asked about the percentage of morseths that has the property in question. The average prevalence that led participants to accept generic statements (*M* = 69.1%) was significantly lower than the average prevalence implied by them (*M* = 95.8%). No such difference was found in the “most” condition. With respect to item type, acceptance in the generic condition was significantly higher for the dangerous/distinctive items (68%) than for the neutral items (55%) or the non-distinctive control items (48%). The effect was particularly strong at the lower percentage levels (10 and 30% prevalence levels). In the *most*-condition, participants were not sensitive to this information and gave similar responses to all items (47% for the dangerous/distinctive, 46% for the neutral and 45% for the non-distinctive controls items). Cimpian et al. (2010) conclude that the danger/distinctiveness information had an effect on generics’ acceptability, seen especially at the lower prevalence levels, while it had no effect on items that lacked this feature.

On the interplay between strikingness and exception tolerance, Leslie (2008, p.15) argues that “the criteria that govern troublesome generics reflect our psychology … the more striking, appalling, or otherwise gripping we find the property predicated in the generic, the more tolerant the generic is to exceptions”.

The only attempt to go beyond familiar and high-frequency properties (e.g., sleeps, drinks milk) in studies of the acquisition of generalizations is Graham et al. (2016), who investigated 30-month-olds’ willingness to extend atypical properties to members of an unfamiliar category by asking them to imitate an act. The properties were introduced in one of the following three ways: (a) with a generic noun phrase (“Blicks drink ketchup”), (b) with a non-generic noun phrase (“These blicks drink ketchup”) or (c) with an attentional phrase (“Look at this”). Willingness to extend was boosted by the presence of a generic noun phrase compared to the other two conditions. This shows that atypical properties are also consistently extended when they are presented with a generic nominal. However, this study did not combine typical and atypical properties within the same design, so the difference that property type can make on extension rates was not measured.

There is little, therefore, if any at all, research on what children know about generalizations with striking properties, whether they treat them differently from other types of generalizations, whether varying the number of exceptions prevents them from generalizing and how exception tolerance interacts with the content of a generalization. In our studies we wanted to address the developmental course of different types of generalizations and to establish whether certain generalizations are learned earlier than others. Instead of measuring acceptance rates for striking generalizations directly, we decided to adopt a design that would measure how likely people (both children and adults) are to extend striking information as opposed to neutral information to more instances of the same kind. This design was adopted because it was deemed to be more child-friendly than a truth-value judgment task and because we could rely on pre-existing literature that had developed a task that fit our purposes (Chambers et al., 2008).

### Exceptions

The most distinctive characteristic of generic statements is arguably the fact that they tolerate exceptions (Krifka et al., 1995). The generic generalization “birds fly” can be truthfully uttered even in the face of exceptions, such as flightless birds like penguins or ostriches, whereas the universal generalizations “all birds fly” or “every bird flies” are false given the existence of exceptions such as the above. The percentage of exceptions allowed seems to depend on the type of generic generalization in question ranging from 0%, as in (1), to a few abnormal cases, as in (2), to around 50%, as in (3), and even reaching 99%, as in (4). That is, exception tolerance seems to be a matter of degree and to further depend on the domain in question and the properties predicated of the kind (see discussion in Pelletier, 2010; Leslie et al., 2011).

(1) Ants are insects.
(2) Tigers have stripes.
(3) Ducks lay eggs.
(4) Sharks attack people.

This complex picture is also seen in different acceptance rates for different types of generic generalizations in experimental studies with adults. Leslie et al. (2011, Experiment 1) found that acceptance rates for bare plural generic statements in a forced choice judgment task varied as follows: “quasi-definitional” like (1) were accepted at 90%, “majority characteristic” like (2) at 96%, “minority characteristic” like (3) at 85% and “striking” like (4) at 77%. This experiment though did not explicitly manipulate the exceptions, so we can only infer from the different acceptance rates that they might be due to different degrees of exception tolerance. Tolerance of exceptions was investigated by Lazaridou-Chatzigoga et al. (2019) in an experiment where participants judged the felicity of bare plural generic statements after a short context was provided. There, acceptance rates of majority characteristic generics (e.g., “tigers have stripes,” “horses have four legs”) were lower (87%) when preceded by a context that made accidental exceptions (e.g., albino tigers or three-legged horses) salient, than when a preceding context did not mention any exceptions (99%). By comparison, the acceptance rates for universally quantified statements (e.g., “all tigers have stripes,” “all horses have four legs”) dropped from 81% in the neutral context to only 48% in the context with salient exceptions. The high acceptance rate for generic statements even immediately following a context making exceptions salient confirms that adults are highly tolerant of exceptions to generic statements.

There is some indicative evidence that children know that generics tolerate exceptions (Hollander et al., 2002; Gelman and Raman, 2003; Gelman and Bloom, 2007; Chambers et al., 2008). In research involving familiar kinds and properties, Hollander et al. (2002) found that 4-year-olds (but not 3-year-olds) behaved like adults in that they answered “yes” to generic questions referring to “narrow-scope” properties like “do girls have curly hair?” more often than “all” questions (“do all girls have curly hair?”) but significantly less often than to “some” questions (“do some girls have curly hair?”). Thus, Hollander et al. (2002) concluded that by 4 years of age children treated generics as different from both “some” and “all,” showing that they understand that they allow for the possibility of counterexamples. Gelman and Raman (2003) found that children seem to know that familiar kinds like birds and dogs can have exceptions, such as penguins and three-legged dogs. Their 4-year-old participants performed similarly to adults and answered “yes” to a question like “Here are two birds. Now I am going to ask you a question about birds. Can birds fly?” when they were shown two exceptional birds (in this case, two penguins), while they answered mainly “no” when the question was phrased with a definite (“Can the birds fly?”).

Other research investigates children’s reasoning about novel kinds. When learning about observable properties of novel kinds, Gelman and Bloom (2007) argue that 4- and 5-year-olds recognized that generics can be true despite salient exceptions. They exposed children and adults to scenarios involving novel animals, such as “dobles”, that do or do not have claws. Their results show that under certain contexts both children and adults accepted generic statements such as “dobles have claws” even when not all the specific instances available exhibited the relevant property, i.e., some were claw-less, while they rejected statements with possessives (“my dobles” for the adults) and demonstratives (“these dobles” for the children) in the same situations. Chambers et al. (2008) went one step further and studied abstract properties of novel kinds. In their Experiment 2, after having been introduced to novel kinds (“These are pagons. Pagons are friendly.”), children extended the relevant property to a new instance of the same kind even in the face of a counterexample (i.e., an unfriendly pagon). Extension rates were at 65% with generic bare plurals (“pagons”) but at 27% with a demonstrative (“these pagons”). Only the extension rates after generics were significantly higher than chance.

Overall, the picture that emerges is that by the age of 4 children know that generic generalizations allow for exceptions both with familiar and novel kinds. As discussed above, exception tolerance seems to be a matter of degree and to further depend on the domain in question and the properties predicated of the kind. There is little, if any, research on whether varying the number of presented exceptions for a given generalization would influence how children learn on the basis of generics. The degree of exception tolerance of a given generalization is especially interesting in cases where there seem to be more counterexamples to the rule than positive instances. For instance, for a statement like “sharks attack people,” exceptional sharks that do not attack people are the vast majority of the kind, but people would still accept the statement as true based on the belief/knowledge that a tiny percentage of sharks do so.^1^ Finally, we wanted to investigate how exception tolerance and the type of property interact when children form generalizations.

## The Present Studies

Two experiments were conducted to systematically answer the following three questions: (a) are children sensitive to the content of generalizations in so far as it is not a characteristic but a striking property in the relevant sense? That is, do children extend properties (form generalizations) at the same rate for striking and neutral properties? (b) does varying the number of exceptions have any effect on children’s willingness to extend a property after having heard a generalization? That is, does varying the number of exceptions (presenting many or fewer exceptions) prevent children from generalizing? and (c) if participants are sensitive to the number of exceptions they are presented with when forming a generalization, does the type of property (neutral or striking) make a difference in how the varying number of exceptions affects generalizations? This is a question about the interaction, therefore, of type of property and exception tolerance. In designing the experiments, we relied on Chambers et al. (2008), where novel creatures were introduced to the children, about which they had no prior knowledge. In Experiment 1, we operationalized *strikingness* for children. We asked whether children would show sensitivity to strikingness for generalizations, given that we know that generics are commonly used to convey striking information about kinds. Instead of using examples such as “sharks attack people,” we opted for child-friendly alternatives. In Experiment 2, we asked whether varying the number of exceptions presented would play any role in the participants’ willingness to extend a neutral/striking property to a new instance. Thus, we asked how exception tolerance and the content of generalizations interact.

Based on previous literature showing that 4-year-old understand that generalizations allow for exceptions and that they consistently extend properties to new instances of a novel kind when these are introduced with a generic nominal (Chambers et al., 2008), we predicted that 4- and 5-year-old would show willingness to extend the property. Specifically, we predicted that children would extend the property of a newly presented kind introduced with a generic sentence to a new instance of the kind. We also predicted that adults would behave similarly.

Regarding children’s sensitivity to strikingness, we wanted to investigate whether children know that generalizations can be about different types of properties, ranging from neutral to striking properties. If this sensitivity is established, there is a further issue. If children were aware of the breadth of the claims one can make with a generic, they would be expected to extend both neutral (common, familiar) and striking (uncommon, less typical) properties. The sensitivity to the type of property could take two different directions: (a) it could be that the fact that the property is striking and less common leads participants to be conservative and extend striking properties less than neutral properties, or (b) it could be that both children and adults would extend the property even more in the case of striking properties than with neutral ones in order to maximize transfer of important information about a novel kind. This second direction is linked to Leslie’s theoretical account (2008) and Cimpian et al. (2010)’s empirical findings that showed a stronger willingness to accept a generic when it concerns a dangerous or distinctive property as opposed to a neutral property. On the other hand, support for the first direction comes from the Prasada et al. (2013) finding that adults judged striking generics to be true less often than even “minority characteristic” generics like “ducks lay eggs” in out of the blue contexts where no exceptions are made salient to either type of generalization.

Regarding children’s sensitivity to the degree of exception tolerance as modulated by the type of property in generalizations, our expectations were that it should matter if the property is neutral or striking and, furthermore, whether we have minimal or maximal exceptions. When only one exception was presented (minimal condition), we were expecting lower extension rates than when no exceptions were present, but potentially similarly high extension rates both for neutral and striking properties. When more exceptions were presented (maximal condition), we were expecting lower extension rates than when less exceptions were present (minimal condition). Furthermore, following Leslie (2008) and Cimpian et al. (2010) we were expecting a difference between neutral and striking properties in the maximal condition: the decrease of extension (between minimal and maximal condition) for striking properties should be smaller than the decrease of extension (between minimal and maximal condition) for neutral properties because striking properties are licensed in rare noteworthy cases. That is, the effect of maximal exceptions in the case of striking properties should be weaker given that it is crucially when the property is striking as in the case of “sharks attack people” that we expect that despite the many exceptions, extending the property is still justified. We expected similar behavior with adults.

To summarize, we had the following four predictions (in parentheses we mention the experiment that tests each of them):

*Prediction 1* (Experiment 1) “sensitivity to strikingness”: participants are expected to show sensitivity to strikingness by extending striking properties at a different rate – either more or less – than neutral ones
*Prediction 2* (Experiment 1–Experiment 2): “sensitivity to exceptions”: in the face of exceptions, extensions will be lower than when no exceptions are present for both kinds of properties
*Prediction 3* (Experiment 2): “sensitivity to number of exceptions”: the greater the number of the exceptions, the lower the extension rates, for both kinds of properties
*Prediction 4* (Experiment 2): “tolerance of maximal exceptions”: following Leslie (2008) and Cimpian et al. (2010), in the case of maximal exceptions, the decrease in extension rate for minimal exceptions to striking properties will be smaller than the decrease in extension rate for minimal exceptions to neutral properties, that is, striking generalizations would show a greater tolerance of maximal exceptions

### Experiment 1

In Experiment 1, we operationalized strikingness for children. Chambers et al. (2008) chose imperceptible properties that were either trait terms (*friendly, shy, gentle, mean*) or physical characteristics (*strong, fast*), which were familiar and common. They did not control for noteworthiness or strikingness as they had different purposes in that study. We constructed items with precisely these dimensions of meaning in mind in order to ask whether children would show sensitivity to strikingness for generics. Instead of using examples such as “sharks attack people,” we opted for child-friendly alternatives. We also collected adult data for comparison. We describe the items in more details in the section below.

Both studies were carried out in accordance with the recommendations of the Cambridge Psychology Research Ethics Committee with written informed consent from all subjects. Adult subjects gave written informed consent and parents gave written informed consent for their children to participate in the studies in accordance with the Declaration of Helsinki. The study was approved by the Cambridge Psychology Research Ethics Committee.

#### Method

##### Participants

A total of 32 English-speaking children (18 male; 14 female) between 51 and 69 months of age (*M* = 60.53, *SD* = 5.1) were recruited from a local primary school in London, United Kingdom. All children were English native speakers and residents in London, United Kingdom. The sample included both monolingual and bilingual children. For bilinguals, their parents and teachers assessed that English was their dominant language.

A total of 140 English-speaking adults (69 male, 71 female) between 18 and 66 years of age (*M* = 35.35, *SD* = 11.95) were recruited via Prolific Academic, an on-line recruitment platform for research.^2^ They were all English native speakers and residents in the United Kingdom.

Participants were randomly assigned to one of two conditions: neutral (16 children; 70 adults) and striking (16 children; 70 adults).

##### Design

**FIGURE 1:**
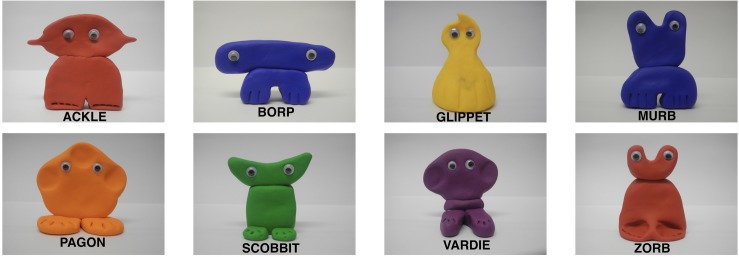
Examples of eight novel kinds used in the experiments: ackle, borp, glippet, murb, pagon, scobbit, vardie, zorb.

The design was based on Chambers et al. (2008) Experiment 2. Eight kinds of novel creatures were created using modeling clay and six instances were created for each kind (only three of which were used in Experiment 1). The individual instances differed only in color, and the experimenter pseudorandomised the color combinations between trials in a way that prevented participants making generalizations on the basis of color similarity. Each creature was given one of eight novel names (*ackle, borp, glippet, murb, pagon, scobbit, vardie, zorb*)^3^ and was paired with one property which was either “neutral” or “striking” in the relevant sense. The creatures can be seen in Figure 1 below.

For the properties, we decided to use imperceptible, non-obvious properties that could not be assessed on the basis of the visual features of our experimental creatures. Instead of using adjectives as Chambers et al. (2008) did, we used verbal predicates and embedded them under the dispositional verb “love” for maximum arousal and in order to make them more child-friendly. In order to identify possible properties, we used affective ratings from Warriner et al. (2013), who measured valence, arousal, and dominance for 13,915 English lemmas by collecting adult ratings on a 9-point scale. The three dimensions were *valence* (the pleasantness of the stimulus), *arousal* (the intensity of emotion provoked by the stimulus) and *dominance* (the degree of control exerted by the stimulus). Examples of the database at the extreme ends include the following: *pedophile* (lowest valence: 1.26), *vacation* (highest valence: 8.53), *grain* (lowest arousal: 1.6), *insanity* (highest arousal: 7.79), *dementia* (lowest dominance: 1.68) and *paradise* (highest dominance: 7.9).

We identified words with high arousal and average valence, which we used as a child-friendly proxy for “striking” and words with average arousal and high valence, which we used as proxy for “neutral.” We then cross-checked these against the Kuperman et al. (2012) database for AoA (Age of Acquisition) of 30,000 English words in order to select only words that our young participants would know. From this set of items, 8 pairs of predicates that differed minimally in their syntactic structure were created. In the final set of items used (see Table 1) striking properties had high arousal (range 4.91–7.24, *mean* 6.08) and average valence (range 2.53–4.68, *mean* 3.68), while neutral properties had average arousal (range 3.14–5.24, *mean* 3.99) and high valence (range 6.09–7.5, *mean* 6.85).^4^

**TABLE 1:** Items used in Experiments 1 and 2.

**Neutral properties**	**Striking properties**
Ackles love to play with toys	Ackles love to play with fire
Borps love to talk to their mothers	Borps love to scare their mothers
Glippets love to run through parks	Glippets love to smash through walls
Murbs love to draw their names	Murbs love to shout their names
Pagons love to feel safe	Pagons love to feel afraid
Scobbits love to make new games	Scobbits love to cheat at games
Vardies love to play with cats	Vardies love to play with snakes
Zorbs love to make people sing	Zorbs love to make people angry

##### Norming study

We performed a norming study online (38 adult English speakers recruited from Prolific Academic took part via Qualtrics) in order to control for baseline preferences for the experimental properties. Participants were introduced to the creatures used in the study in order to create a similar mind-frame to the one used in the experiment and were then asked to judge how likely a given property is, e.g., “how likely are ackles to love to play with toys,” “how likely are ackles to love to play with fire” etc. “Neutral” properties were judged to be more likely on average (*M* = 64.15, *SD* = 12.43) than “striking” properties (*M* = 32.13, *SD* = 11.84). We found a significant difference between “neutral” and “striking” properties, as expected (*t* = 5.2766, df = 13.967, *p* < 0.001).

##### Procedure and materials

Children were tested individually in a quiet room in their school. They were told that they would play a game with a puppet, called Sarah. Sarah, whose voice was recorded by a female English native speaker from the United Kingdom, presented the stimuli and asked questions. During the pretest, familiar objects were used in order to make sure that each child could accurately understand and respond to questions about members of a category (e.g., judging whether different objects that were presented one after the other could be called a “pig” – the objects used were a penguin, a pig and a monkey). After each question in the pretest, feedback was given to the child to make sure that they understood how to model their replies (by answering either “yes” or “no”) to the question asked. No feedback was given during the main task. On each trial of the main task, the following procedure was followed, after which the child’s response to the question was recorded and the next trial began. The mean duration of the whole procedure for children was 8 min 43 s (range: 7 min 35 s–11 min 30 s). Each trial included the following two steps:

Step 1: Two instances of a novel creature were introduced by Sarah (pre-recorded voice) and a property was attributed using a generic nominal: “These are ackles. Ackles love to play with toys (neutral)/fire (striking).”
Step 2: A third instance of the same kind was presented along with a question that measured extension of the property: “Does this ackle love to play with toys (neutral)/fire (striking)?”

Adults completed an equivalent task embedded in a webpage (Qualtrics). The adult version used the same script with a story about a researcher substituting for the puppet in the live action version, and with pictures of the novel kinds. Instead of listening to a pre-recorded voice, the adults read the sentences attributed to the researcher speaking. The task was the same, that is, participants answered the question that measured extension of the property to a new instance of the same kind by pressing the corresponding key for “yes” or “no.” The mean duration of the whole procedure for adults was 6 min and 11 s (range: 3 min 22 s–25 min 18 s).

#### Results and Discussion

**FIGURE 2:**
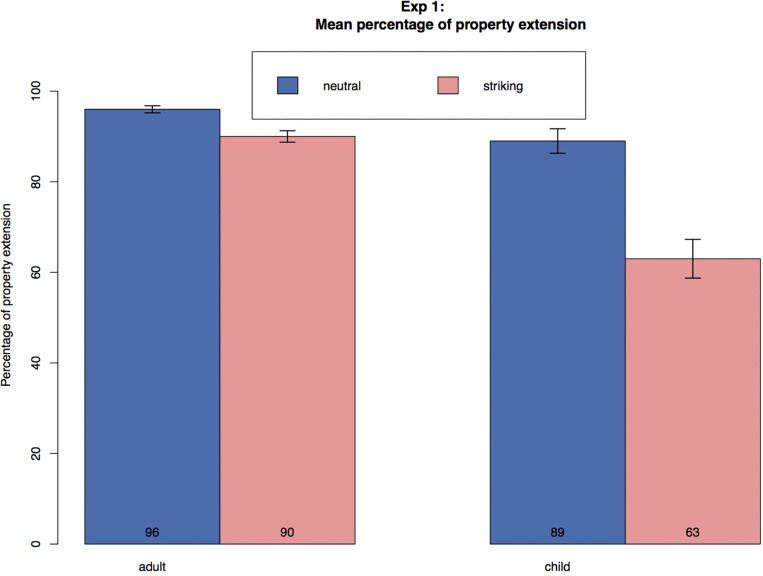
Mean percentage of property extension across conditions in Experiment 1. Error bars represent standard error.

Children extended both types of properties, with the neutral properties giving rise to more extensions (89%) than the striking ones (63%), while adults’ mean extension rates for neutral properties were at 96% and for striking properties at 90% (see Table 2 and Figure 2 below).

**TABLE 2:** Mean percentage of property extension across conditions in Experiment 1.

		**Adult**	**Child**
Experiment 1	Neutral	96.43% (SE 0.78)	89.06% (SE 2.76)
	Striking	90.18% (SE 1.26)	63.28% (SE 4.28)

*Standard error in parenthesis.*

We used R (R Core Team, 2016) and the lme4 package (Bates et al., 2015) to perform a generalized linear mixed-effects analysis, specifying a binomial family. First, we fitted a model including all of the relevant variables, that is, property.type and age (a binary factor, child vs. adult) as fixed effects (with an interaction term) and subject and item.no as random effects. Then, we performed likelihood ratio tests. If the variable was part of an interaction, we first tested the model with the interaction against a model with just the main effects. The comparison proved non-significant (χ^2^(1) = 1.5705, *p* = 0.21). Thus, adding an interaction term did not significantly improve model fit, so we used the model without the interaction term for all subsequent analyses. The full model parameters (without the interaction) are provided in Table 3.

**TABLE 3:** Estimates, standard errors, *z* values and *p* values of the full glmm in Experiment 1.

	**Estimate**	**Std. Error**	***z* value**	**Pr(< |z|)**
Intercept (adult, neutral)	6.5829	0.9515	6.919	4.56e-12^∗∗∗^
Age (child)	–2.2553	1.0084	–2.237	0.0253^∗^
Property (striking)	–1.8216	0.7791	–2.338	0.0194^∗^

*Significance codes: ^∗∗∗^ 0.001, ^∗∗^ 0.01, ^∗^ 0.05.*

We then tested the main effects by fitting versions of the full model, from which a single effect was removed, and then compared the reduced model to the full model. In order to test the main effect of property, we removed property.type. We performed a likelihood ratio test of the full model against the model without property.type and the comparison proved significant (χ^2^(1) = 4.7778, *p* = 0.029). Thus, we concluded that there was a main effect of property in that striking properties were extended less than neutral properties. In order to test the main effect of age, we removed age. We performed a likelihood ratio test of the full model against the model without age and the comparison proved significant (χ^2^(1) = 4.264, *p* = 0.039). Thus, we concluded that there was a main effect of age in that children were less likely to extend than adults in both conditions.

Additional analyses were performed in order to determine whether the percentage of property extensions was significantly greater than chance. In order to do so, we fitted a glmer model with only an intercept, no fixed predictors and subject as a random effect. The intercept is the overall average property extension rate and given that there were only two choices (“yes”/“no”), zero corresponds to 50% extension (chance levels are at 50%), so we can see if the intercept is significantly different from that. The intercept was significantly different (β = 7.52, SE = 0.84, *z* = 9, *p* < 0.001) from chance, thus we conclude that the performance overall was significantly different from chance. Then, we fitted a glmer model without an intercept in order to look into individual conditions: the responses to both neutral and striking properties were significantly different from chance (neutral: β = 7.82, SE = 0.99, *z* = 7.87, *p* < 0.001; striking: β = 6.32, SE = 1.1, *z* = 5.75, *p* < 0.001). Finally, we fitted a glmer model without an intercept in order to look into the different age groups; the responses of both adults and children were significantly different from chance (adult: β = 7.39, SE = 0.94, *z* = 7.85, *p* < 0.001; child: β = 5.58, SE = 1.54, *z* = 3.63, *p* < 0.001).

The final analysis performed involved comparing the results of Experiment 1 to the norming study. Focusing only on the adults, we observe that acceptance for both conditions significantly increased in Experiment 1 (neutral: 96%; striking: 90%) compared to the baseline measures of the norming study (neutral: 64%; striking: 32%) (*t* = 8.6808, df = 16.598, *p* < 0.001). We attribute this increase to the use of generic linguistic forms in Experiment 1 as opposed to asking general questions about the properties in the norming study.

#### Discussion

This study serves as the first experiment that operationalizes strikingness in the acquisition of properties of novel kinds via generic generalizations. First, given that we obtained a main effect of property, we conclude that both adults and children show sensitivity to strikingness. This confirms that the content of a generalization is important when learning about novel kinds and when drawing inferences based on the type of property used. The direction of the effect shows that both groups extended striking properties less than neutral ones. It is also worth highlighting that even the reduced acceptance rate for striking properties was above chance for both children and adults. That is, children are not only willing to extend a property to a new kind when it is a neutral one like “playing with toys,” but also when it is a striking one like “playing with fire.” That means that strikingness does not prevent adults or 4- and 5-year-old children from being willing to extend properties that they learned via generic generalizations. The finding that both adults and children extended striking properties less than neutral ones hasn’t been documented before in the literature and calls for an explanation which we attempt in section “General Discussion.” The fact that we observe the same effect for both children and adults suggests that whatever it is that makes striking generalizations different from neutral generics is already relevant to 4- and 5-year-old children.

Second, given that we obtained a main effect of age, we conclude that the children have not yet reached fully adult-like willingness to license a generic inference given the type of property. Mean extension rates across conditions for adults are very high (for both conditions more than 90%) and higher than the children’s mean extension rates. That is, we observe a developmental effect in the way children treated neutral/striking properties of novel kinds, and specifically that they were more conservative than adults when it came to extending the property to new instances of the novel kind. However, based on the analyses on the difference from chance level, we conclude that both adults and children performed in the task in a statistically meaningful way.

In Experiment 2 we build on these results and seek an explanation for the reduced extension rates of striking properties compared to neutral properties while we address the issue of whether children’s and adults’ sensitivity to the type of property is modulated by varying the number of exceptions.

### Experiment 2

In Experiment 2, we asked whether the number of exceptions introduced would play any role in the participants’ willingness to extend a neutral/striking property to a new instance of a novel kind. We expected lower extension rates than in Experiment 1 due to the presence of exceptions and a sensitivity to the number of exceptions, that is, we expected that the greater the number of exceptions, the lower the extension rates would be. Finally, we expected that striking properties would have a greater tolerance of maximal exceptions than neutral ones (see section “General Discussion” for a discussion on our predictions).

#### Method

##### Participants

A total of 32 English-speaking children (20 male; 12 female) between 52 and 70 months of age (*M* = 60.29, *SD* = 5.13) were recruited from two local primary schools in London, United Kingdom. All children were English native speakers and residents in London, United Kingdom. The sample included both monolingual and bilingual children. For bilinguals, their parents and teachers assessed that English was their dominant language.

A total of 152 English-speaking adults (78 male, 74 female) between 18 and 74 years of age (*M* = 38.57, *SD* = 12.94) were recruited via Prolific Academic, an on-line recruitment platform for research. They were all English native speakers and residents in the United Kingdom.

Participants were randomly assigned to one of four conditions: neutral-minimal (8 children; 38 adults), neutral-maximal (8 children; 38 adults), striking-minimal (8 children; 38 adults) and striking-maximal (8 children; 38 adults).

##### Design

###### Procedures and materials

The procedure and the materials were identical to the ones used in Experiment 1 except for the addition of an intermediate step that involved introducing varying numbers of exceptions (1 in the minimal, 3 in the maximal condition). As in Experiment 1, materials were presented to children via pre-recorded sound files acted out by an experimenter using Sarah the puppet. The mean duration of the whole procedure for children was 10 min 16 s (range: 7 min 50 s–13 min 30 s). Adults saw the scenarios accompanied by written descriptions on a computer screen. The mean duration of the whole procedure for adults was 7 min 59 s (range: 2 min 40 s–19 min 55 s). Each trial included the following three steps:

Step 1: Two instances of a novel creature were introduced and a property was attributed using a generic nominal: “These are glippets. Glippets love to play with toys (neutral)/fire (striking).”
Step 2: One/Three instances of the same kind was/were shown and identified as an exception/exceptions to the previous assertion: “But not this one. This glippet doesn’t love to play with toys/fire (minimal)/But not these ones. These glippets don’t love to play with toys/fire (maximal).”
Step 3: A fourth/sixth instance of the same kind was presented along with a question that measured extension of the property: “Does this glippet love to play with toys/fire?”

#### Results and Discussion

**FIGURE 3:**
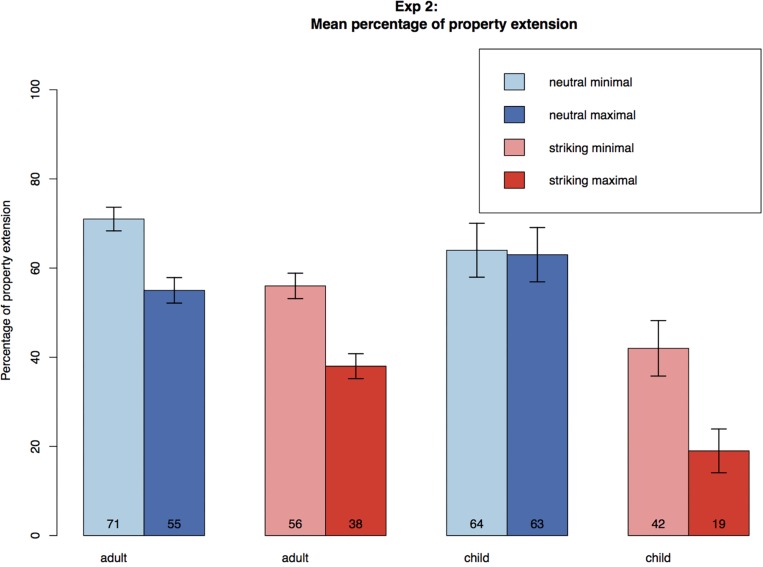
Mean percentage of property extension across conditions in Experiment 2. Error bars represent standard error.

Both adults and children extended neutral and striking properties even in the face of exceptions. For adults in the neutral condition, extension rates were higher in the minimal (71%) than in the maximal condition (55%). In the striking condition, extension rates were lower than in the neutral condition: they were at 56% for the minimal and 38% for the maximal condition. For children in the neutral condition, extension rates were similar in the minimal (64%) and in the maximal condition (63%). In the striking condition, extension rates were lower than in the neutral condition, they were at 42% for the minimal and at 19% for the maximal condition (see Table 4 and Figure 3 below). Overall, striking properties gave rise to fewer extensions that neutral ones.

**TABLE 4:** Mean percentage of property extension across conditions in Experiment 2.

		**Adult**	**Child**
Experiment 2	Neutral minimal	71.18% (SE 2.65)	64.06% (SE 6.05)
	Neutral maximal	54.60% (SE 2.86)	62.50% (SE 6.09)
	Striking minimal	55.60% (SE 2.85)	42.18% (SE 6.22)
	Striking maximal	38.16% (SE 2.79)	18.75% (SE 4.91)

*Standard error in parenthesis.*

First, we fitted the full model with property.type, exception.type and age as fixed effects (with a three-way interaction term) and subject and item.no as random effects. After building the full model, we built a model without the three-way interaction, that is, a model with all two-way interactions. We performed a likelihood ratio test of the full model with the three-way interaction term against the model without the three-way interaction and the comparison proved non-significant (χ^2^(1) = 1.337, *p* = 0.248). Including the three-way interaction term did not significantly improve model fit, so we used the model without the three-way interaction term for all subsequent comparisons. We then built subsequent models by removing one two-way interaction at a time (property.type:exception.type, property.type:age, exception.type:age). We performed a likelihood ratio test of the model without the three-way interaction term against the model without the property type:exception.type interaction and the comparison proved non-significant (χ^2^(1) = 0.1747, *p* = 0.676). Then, we performed a likelihood ratio test of the model without the three-way interaction term against the model without the property.type:age interaction and the comparison proved non-significant (χ^2^(1) = 3.2976, *p* = 0.069). Then, we performed a likelihood ratio test of the model without the three-way interaction term against the model without the exception.type:age interaction and the comparison proved non-significant (χ^2^(1) = 0.1108, *p* = 0.739). Finally, we built a model with the same fixed and random effects, but without any interaction terms. We performed a likelihood ratio test of the full model with the three-way interaction term against the model without any interaction terms and the comparison proved non-significant (χ^2^(4) = 4.9706, *p* = 0.29). Thus, adding interaction terms did not significantly improve model fit, so we used the model without any interaction terms for all subsequent analyses/comparisons. The full model parameters (without any interaction) are provided in Table 5.

**TABLE 5:** Estimates, standard errors, *z* values and *p* values of the full glmm in Experiment 2.

	**Estimate**	**Std. Error**	***z* value**	**Pr(< |z|)**
Intercept				
(adult, neutral, minimal)	0.04528	0.20245	0.224	0.8230
Age (child)	–0.45459	0.26177	–1.737	0.0825
Property (striking)	–1.02026	0.19664	–5.189	2.12e-07^∗∗∗^
Exception (maximal)	–0.87056	0.19588	–4.444	8.82e-06^∗∗∗^

*Significance codes: ^∗∗∗^ 0.001, ^∗∗^ 0.01, ^∗^ 0.05.*

We then fitted versions of this full model, from which a single effect was removed and compared the reduced model to the full model. In order to test the main effect of property, we removed property.type. We performed a likelihood ratio test of the full model against the model without property.type and the comparison proved highly significant (χ^2^(1) = 25.985, *p* < 0.001). Thus, we concluded that there was a main effect of property in that striking properties were extended less than neutral properties. In order to test the main effect of exception, we removed exception.type. We performed a likelihood ratio test of the full model against the model without exception.type and the comparison proved highly significant (χ^2^(1) = 19.043, *p* < 0.001). Thus, we concluded that there was a main effect of exception in that minimal exceptions gave rise to more extensions than maximal exceptions. In order to test the main effect of age, we removed age. We performed a likelihood ratio test of the model without interactions against the model without age and the comparison did not prove significant (χ^2^(1) = 2.98, *p* = 0.084). Thus, we concluded that there was no main effect of age.

Additional analyses were performed in order to determine whether the percentage of property extensions was significantly greater than chance. In order to do so, we fitted a glmer model with only an intercept, no fixed predictors and subject and item.no as random effects. The intercept is the overall average property extension rate and given that there were only two choices (“yes”/“no”), zero corresponds to 50% extension (chance levels are at 50%), so we can see if the intercept is significantly different from that. The intercept was not significantly different (β = 0.19, SE = 0.19, *z* = 1.03, *p* = 0.302) from chance, thus we concluded that the performance overall was not significantly different from chance. Then, we fitted a glmer model without an intercept in order to look into individual conditions (collapsed across the two age groups); the responses to neutral properties in the minimal condition (β = 1.1, SE = 0.26, *z* = 4.3, *p* < 0.001) and to striking properties in the maximal condition (β = –0.79, SE = 0.25, *z* = –3.15, *p* = 0.002) were significantly different from chance, while responses to neutral properties in the maximal condition (β = 0.31, SE = 0.25, *z* = 1.26, *p* = 0.21) and to striking properties in the minimal condition (β = 0.16, SE = 0.25, *z* = 0.66, *p* = 0.51) were not significantly different from chance. Finally, we fitted a glmer model without an intercept in order to look into the different age groups; only the adults’ responses were significantly different from chance (β = 0.25, SE = 0.12, *z* = 2.16, *p* = 0.03) and children’s responses were not significantly different from chance (β = –0.19, SE = 0.26, *z* = –0.74, *p* = 0.46).

Comparing extension rates between the two experiments, we confirm that extension rates are lower in the presence of exceptions (Experiment 2) as compared to when no exceptions are discussed or made salient (Experiment 1) for any type of property and in both age groups. In an additional analysis that combined the results of both experiments in order to address the issue of whether the presence of exceptions had any effect on the results, we were able to determine that the presence of exceptions significantly lowered the rate of extensions (*p* < 0.001).

#### Discussion

Experiment 2 manipulated the number of exceptions in order to see how it would affect extension of different types of generics (neutral/striking). First, we observe that in both groups, extension occurs with both types of generics despite the presence of exceptions. Given that we obtained a main effect of property, we observe that in both groups there is a sensitivity to strikingness. We confirm that striking properties are extended less than neutral properties, as in Experiment 1. Second, by comparing extension rates between the two experiments, we confirm that extension rates are lower in the presence of exceptions (Experiment 2) as compared to when no exceptions are discussed or made salient (Experiment 1) for any type of property and in both age groups. Third, given that we obtained a main effect of exception, we observe that the number of exceptions influences extension rates. For both types of property in adults, the greater the number of exceptions, the lower the rates of extension obtained. The picture was different for children though, where the number of exceptions influenced only striking properties. Fourth, maximal exceptions had the same effect on both neutral and striking properties in adults, that is, they lowered extension rates as compared to minimal exceptions. Thus, we did not observe a higher tolerance of maximal exceptions for striking properties. In children, maximal exceptions lowered extension rates for striking properties, but not for neutral properties. Finally, given that we obtained no main effect of age, it seems that children are already exhibiting behavior within the adult range. The overall distribution of child responses seems to overlap with the overall distribution of adult responses. Nevertheless, as the analyses on the difference from chance level showed, even in the absence of a main effect of age, the child behavior was very variable suggesting that the children’s performance could still be chance performance.

## General Discussion

The two experiments discussed here are the first studies to test the strikingness of the property as a factor in forming generalizations. The first study demonstrates that children know that generics tolerate exceptions and that they are sensitive to the content of the generalization presented. 4- and 5-year-olds effectively use generic language in order to draw inferences about new instances of a novel kind and they are adult-like in that they extend striking properties less than neutral properties. Nevertheless, they have not yet reached adult-like behavior.

A sensitivity to the content of the generalization was found in both adults and children. The inferences drawn, and the willingness to extend the property to a new exemplar of a novel kind depended on whether the generalization concerned something that is considered neutral in the relevant sense, that is, something common or ordinary, or something that is considered striking in the relevant sense, that is, something unusual or remarkable. Related to prediction 1 above (“sensitivity to strikingness”), we had hypothesized two possible outcomes for the direction of this sensitivity, repeated here for ease of reference: (a) it could be that the fact that the property is striking and less common leads participants to be conservative and extend striking properties less than neutral properties as would be consistent with the Prasada et al. (2013) findings, or (b) it could be that both children and adults would extend the property even more in the case of striking properties than with neutral ones in order to maximize transfer of important information about a novel kind. This second direction seems linked to Leslie’s theoretical account (2008) and Cimpian et al. (2010)’s empirical findings that showed a stronger willingness to accept a generic when it concerns a dangerous or distinctive property as opposed to a neutral property. The observed sensitivity in our two studies supports the first direction. That is, our results confirm that striking properties are extended less than neutral properties by both age groups.

There are two possible explanations for the fact that our results are consistent with the “conservative” option. The first one is a methodological feature and the second one is related to how we operationalized strikingness.

The first explanation concerns methodology. Our task involved measuring extension to a new exemplar of a novel kind while Cimpian et al. (2010)’s task measured whether a given statement about a novel kind was true or false. Our task was also more immersive given the more naturalistic environment we adopted: in our study, participants could see and observe the novel kinds, whereas in Cimpian et al. (2010)’s study (adult) participants only read statements about the novel kinds. It is not clear to us at this stage how these different designs might have contributed to the different pattern of results, but the point we want to make here is that the tasks were methodologically different.

The second explanation concerns the way we operationalized strikingness. In an attempt to construct child-friendly striking properties, strikingness was only indirect or implicit in our study. That is, our participants had to infer the potential danger/distinctiveness from the little information they were given. This does not mean though that our participants did not understand the difference between neutral and striking properties. Our results confirmed that even this implicit way of operationalizing strikingness was enough to trigger different responses by both adults and children. On the contrary, Cimpian et al. (2010)’s items were much longer (see above) and the danger/distinctiveness dimension was made salient through explicit mention of “danger” and description of the potential harm in graphic terms or detailed reference to distinctiveness (by mentioning that no other animal has that feature). This leads us to speculate that in order to get the effect that Cimpian et al. (2010) obtained (that is, that the danger/distinctiveness information increased acceptance rates for generics, especially at the lower prevalence levels) the items should operationalize strikingness in explicit terms.

The second study confirms the finding that both adults and children extend striking properties less than neutral properties and shows that in the presence of exceptions both adults and children extend less overall. Thus, prediction 2 (“sensitivity to exceptions”) was also borne out. The explicit mention of exceptions made both adults and children generalize less, a behavior that has been also observed in other studies with adults (Lazaridou-Chatzigoga et al., 2019). Furthermore, adults are not only sensitive to the presence of exceptions, but also to the number. When more exceptions were present, we observed lower extension rates with both age groups with striking properties, but with neutral properties this was the case only with adults. That is, children are sensitive to the number of exceptions only when the generalization concerns a striking property. Thus, prediction 3 (“sensitivity to number of exceptions”) was only partly confirmed. The reason why varying the amount of exceptions did not alter children’s willingness to extend a neutral property remains to be further investigated. It could be that for neutral properties children need exposure to more instances of a kind to be able to appreciate the relevance of exceptions.

It is not clear though how children integrate these two features of a generalization together, that is, the type of property and the amount of exceptions, as their behavior does not differ from chance in Experiment 2. Thus, even though superficially children seem to demonstrate the ability to accommodate varying degrees of exceptions for striking properties while learning about new kinds and to keep track of the amount of exceptions presented to the generalization in question, we cannot securely attribute this to a learned behavior in the same way that we can with adults.

We had furthermore hypothesized that striking properties would show increased tolerance of maximal exceptions for both age groups (prediction 4, “tolerance of maximal exceptions”). This prediction was not borne out; indeed, we find the opposite pattern, with less tolerance of extensions for striking generalizations than neutral generalizations in both the minimal and maximal conditions. Given the above discussion of the Cimpian et al. (2010) study, it may be that in order for “strikingness” to license generalizations in the absence of statistical prevalence, it has to be clear that the property constitutes a threat to others as in the case of our item “playing with fire.” Some of our items might be dangerous or threatening just for the creature that is involved in the activity in question, but do not necessarily pose a threat to others e.g., “playing with snakes.” Future research can help clarify this point.

### Future Research

In future research, we aim to build on the results of the present experiments. More work on the licensing conditions of strikingness for adults is needed given that our results diverged from other results in the literature. Using versions of our materials that make explicit mention of danger/distinctiveness only with adults in the first instance would help determine whether the way we operationalized strikingness affected the results. The subsequent challenge would be to prepare versions of these explicit materials without being too alarming in order to be able to use them to test young children. An additional interesting dimension of strikingness is the fact that even though the literature has focused on *negative* striking properties that concern dangerous or alarming behavior, the same could arguably hold for *positive* striking properties (which could concern for instance people helping save someone else’s life or making large donations). As Leslie (2017) notes, such cases are not as straightforward and it is not easy to come up with examples. It would be interesting to test, however, whether positive striking properties would receive similar extension rates to negative striking properties as it would help address the question of how essential the dimension of danger or threat is for striking properties.

Other directions for future research would be to establish whether property extension was influenced not only by *what* evidence was presented to the children, but also by *how* evidence was presented, as a recent study (Lawson, 2017) suggests that presenting evidence exemplars at the same time (i.e., simultaneous presentation) or one by one (i.e., sequential presentation) influenced property projections. Moreover, it would be worthwhile to explore how our items relate to a finding in the literature that relates to children’s and adults’ intuitions about the balance between within-category homogeneity and variability in induction (Brandone, 2017). For example, individual cats may differ considerably in color but also on other transient properties including being asleep or sick. Brandone (2017) suggests that children begin to differentiate their inference making based on conceptual knowledge about the nature of the property they are asked to generalize and the category domain by 6–8 years age. Thus, further experiments with older children could reveal how children’s inference making about novel kinds is influenced by such factors.

In sum, the findings underscore that 4- and 5-year-old children are sensitive to the type of property and to exception tolerance when they are making generalizations and are developing toward an adult-like behavior with respect to the interplay between strikingness and exception tolerance when they learn about novel kinds.

## Data Availability

The datasets generated for this study are available on request to the corresponding author.

## Ethics Statement

These studies were carried out in accordance with the recommendations of the Cambridge Psychology Research Ethics Committee with written informed consent from all subjects. Adult subjects gave written informed consent and parents gave written informed consent for their children to participate in the studies in accordance with the Declaration of Helsinki. The protocol was approved by the Cambridge Psychology Research Ethics Committee.

## Author Contributions

DL-C recruited participants, conducted the studies both at schools and online, performed the statistical analysis, and wrote the first draft of the manuscript. NK and LS wrote sections of the manuscript. All authors contributed to conception and design of the studies as well as preparation of the experiments and construction of stimuli, revised the manuscript, and read and approved the submitted version.

## Conflict of Interest Statement

The authors declare that the research was conducted in the absence of any commercial or financial relationships that could be construed as a potential conflict of interest.

## Footnotes

1 To put things into real world perspective, according to the Florida Museum of Natural History’s Department of Ichthyology there is a worldwide average of 50–70 shark attacks every year. Humans, in contrast, annually kill approximately 100 million sharks (Worm et al., 2013).

2 For both experiments, the sample size for adults was at least three times larger than the sample size for children. This decision was based on research that compares Internet-based data to lab-based data, which has shown that the data quality is similar as long as the Internet-based sample size is at least three times larger than the lab-based sample size (see Gosling et al., 2004).

3 For the creatures’ names, we created a list of names used in previous studies (Chambers et al., 2008; Cimpian et al., 2010; Gelman et al., 2015) and normed them with English native adult volunteers from the United Kingdom to check which ones sounded more natural in both the singular and the plural form and more different from existing words. From the higher ranked ones, we chose eight, some mono- and some disyllabic, and we made sure they started with different sounds and included a range of vowel and consonant sounds.

4 Our initial aim had been to use words with average arousal and average valence for “neutral” properties. This was not possible though due to the restricted set of properties that were both known to our young participants according to the AOA database and would help us keep the syntactic structure of our items as close as possible between neutral and striking properties. The choice was therefore made to include items with high valence, which contrast clearly with the striking items.
